# Early Depression Detection in Social Media: Monitoring of Individual Nighttime Dynamics and Large Language Model Analysis

**DOI:** 10.2196/87138

**Published:** 2026-05-29

**Authors:** Bicheng Yu, Zhichang Zhang, Lulu Ma, Jiongfu Cai, Yuanyuan Zhang

**Affiliations:** 1 College of Artificial Intelligence and Computer Science Northwest Normal University Lanzhou, Gansu China

**Keywords:** depression, early risk detection, sleep analysis, large language model, circadian rhythm

## Abstract

**Background:**

Depression has become a major global public health challenge, and early intervention is critical for improving patient outcomes. Current depression detection techniques based on social media data (traditional risk detection) rely heavily on users’ complete historical information, which cannot meet the timeliness requirements of early intervention. This underscores the need for early risk detection (ERD) methods emphasizing early-stage, real-time warning. However, existing ERD studies have notable limitations such as (1) they overlook temporal activity patterns hidden in posting time stamps, missing vital warning signals; and (2) they depend on static templates or resource-intensive sequence models, resulting in limited interpretability and inefficient use of early data, ultimately constraining their clinical applicability for early intervention.

**Objective:**

This study aims to develop an efficient, reliable, and interpretable ERD model. The core objectives are to extract temporal activity patterns features from posting time stamps, thereby enriching feature dimensions for risk detection; to leverage large language models (LLMs) for improved text filtering precision and depression-related factor analysis; and to achieve accurate early detection of depression to support clinical intervention.

**Methods:**

We propose the Monitoring of Individual Nighttime Dynamics (MIND) and LLM analysis model, which integrates two key innovations: (1) circadian activity dynamics: posting time stamps are transformed into temporal activity patterns, analyzing fluctuations in posting frequency and timing to derive sleep-related features, thereby compensating for the limitations of text-only approaches, and (2) LLM depression profiler: LLMs are used for dynamic text filtering, automatically removing irrelevant noise and focusing on potential depression-related cues. Based on LLM semantic understanding, latent depression risk factors are identified, enhancing interpretability for clinical treatment and robustness to noise.

**Results:**

Experiments on the eRisk2017 (data source: Reddit [Reddit Inc]) benchmark dataset demonstrated that MIND significantly outperformed existing baseline models in early detection sensitivity, specificity, and accuracy. By combining sleep-related features with text analysis, the model achieved interpretable, traceable predictions that can support clinical treatment. ALL relevant experimental code is publicly available.

**Conclusions:**

The MIND model combines temporal activity pattern features with LLM-based text analysis, addressing the challenges of poor interpretability and inefficient use of early-stage data in existing ERD methods. It significantly enhances early detection performance, offering a new paradigm for applying social media data in ERD, thereby enabling earlier intervention and reducing the public health burden of depression.

## Introduction

### Background

**Figure 1:**
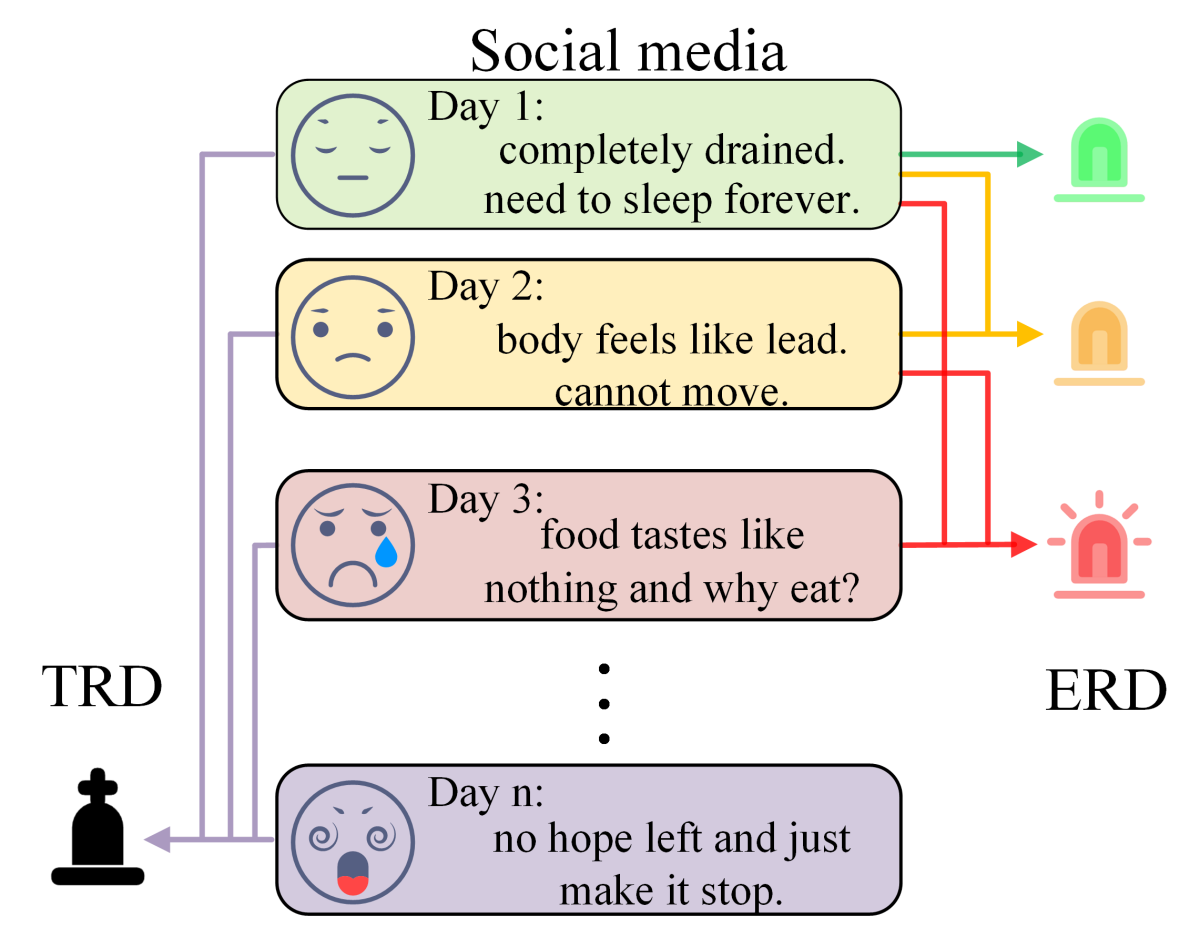
Task schematic diagram. The left side illustrates the traditional risk detection (TRD) task, which predicts a user’s depression status using all available user information. In contrast, the right side represents the early risk detection (ERD) task, which aims to predict a user’s depression status by using early-stage information.

Depression affects approximately 260 million people worldwide [1-3]. This mental disorder leads to prolonged negative emotions, feelings of loss, and, in severe cases, life-threatening risks, making it a major public health concern. As social media increasingly becomes a platform for individuals to record their daily experiences and express emotions, user-generated content often reflects genuine psychological states [4]. This has enabled the academic community to explore social media data for depression detection. Over the years, the eRisk workshop has explored various contexts for identifying psychological disorders and predatory conversations [5-7], contributing valuable insights to the broader field of mental health monitoring through digital traces. Traditional risk detection (TRD) identifies depression from users’ sufficient, long-term social media history and has achieved significant success. However, TRD does not align with the clinical principle of “early detection and early treatment” [8], as it relies on sufficient, long-term user data. To bridge this gap, early risk detection (ERD) has been proposed. ERD aims to predict depression risks based on limited, early-stage user data, allowing timely warnings and interventions. Unlike TRD, ERD prioritizes high precision under constrained data availability, with evaluation criteria focusing not only on final accuracy but also on the earliness of prediction, as illustrated in Figure 1.

### Previous Work

ERD research generally follows two strategies:

1. Long-sequence processing methods: recurrent neural networks [9] and their variants, along with CNNs [10] and variants of the attention mechanism [11], have been used to process full user histories [12-14]. While timestamp data has occasionally been considered, analyses have been limited to simple statistical summaries, failing to capture rhythmic features. Although comprehensive, such methods are resource-intensive and often ineffective at detecting sparse but meaningful emotional signals. Moreover, they cannot identify specific time points of psychological state changes, limiting their clinical value.
2. Filtering and denoising methods: template matching and sliding windows have been used to filter high-information texts [15,16], reducing noise complexity. However, these static methods lack adaptability to individual differences and developmental trajectories. While effective in identifying overt depressive content, they struggle with latent signals due to limitations of earlier NLP methods, offering insufficient support for intervention.

Studies in TRD also continuously provide new insights for the advancement of ERD research. Early TRD approaches relied on text feature analysis using traditional machine learning models (eg, support vector machines and decision trees) and linguistic inquiry and word count–based features [17], but were limited by noise sensitivity and feature unimodality. Multimodal fusion of text, audio, and images [18,19] improved robustness, while more recent studies used large language models (LLMs) for data augmentation and graph structures for multimodal fusion, advancing clinical depression dialogue prediction [20]. Nonetheless, TRD methods remain constrained by their reliance on complete histories, making them unsuitable for early intervention.

In summary, current ERD methods face the following three limitations: (1) neglect of temporal information in time stamps, particularly sleep-related patterns critical to depression; (2) static, template-driven filtering with weak interpretability; and (3) resource-heavy, long-sequence methods that fail to capture sparse but crucial early cues.

### Study Objectives

To overcome these challenges, we propose the Monitoring of Individual Nighttime Dynamics (MIND) model, comprising the following three key modules:

1. Circadian activity dynamics (CAD): by modeling users’ temporal activity patterns from social media posting time stamps and integrating these temporal features with textual information, this module investigates depressive tendencies through the interaction between activity frequency and sleep regularity. This module is grounded in psychological evidence demonstrating a strong association between disrupted sleep patterns and depression. [21-25].
2. LLM depression profiler (LDP): the integration of LLMs enables the automatic filtering of texts from users at high risk of depression and mitigates interference. Building on this, the model analyzes latent negative psychological factors in the text, which enhances information use efficiency and interpretability.
3. Text-sleep fusion (TSF): this module fuses multimodal features (sleep and text) for final early risk predictions.

**Figure 2:**
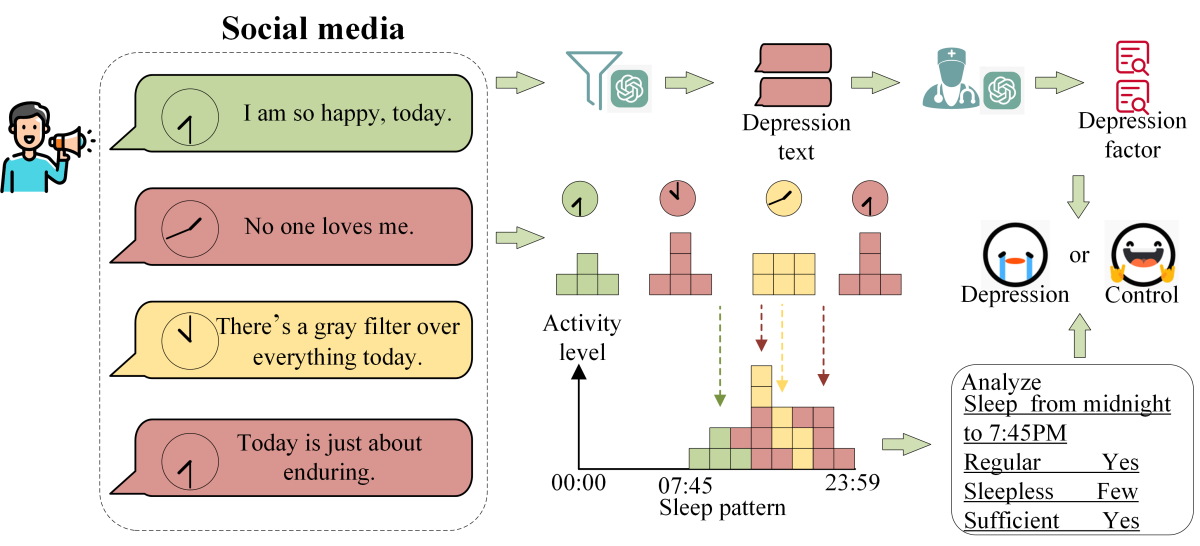
Illustration of innovation points. This figure outlines how user data are transformed into sleep-related features and depression profile features.

**Figure 3:**
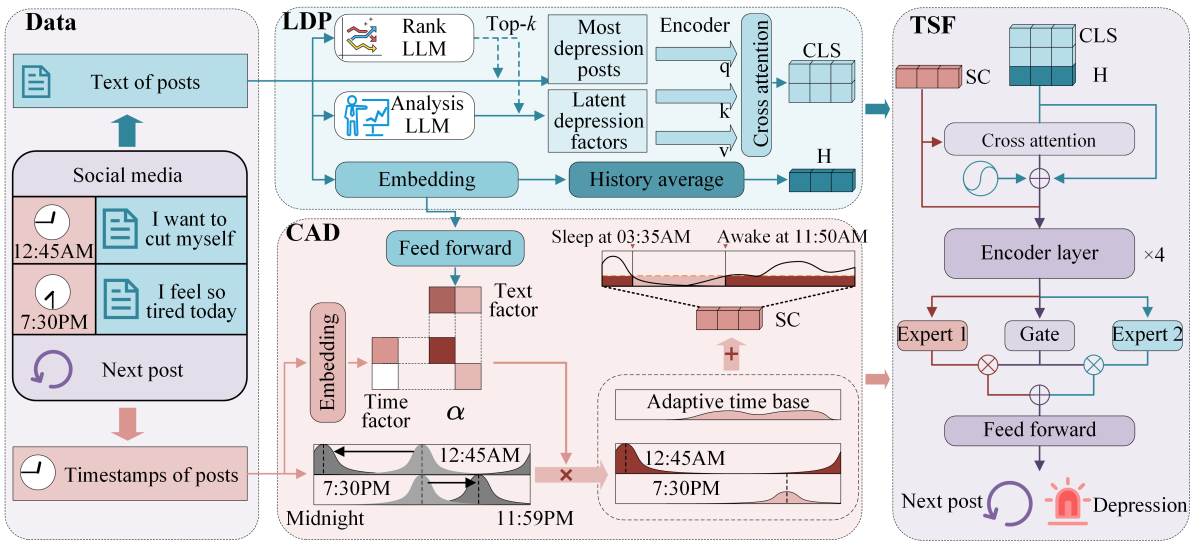
Overview of the Monitoring of Individual Nighttime Dynamics (MIND) model. H: historical representation; LDP: LLM depression profiler; LLM: large language model; SD: sleep dynamics; TSF: text-sleep fusion.

Experiments on the eRisk 2017 dataset validate the effectiveness of MIND. Our contributions include (1) introducing timestamp-based temporal activity patterns inference as an ERD feature; (2) leveraging LLM for text filtering and depression factor analysis to improve efficiency and interpretability; and (3) designing a novel multimodal fusion model (MIND) that significantly enhances early prediction performance. Figure 2 illustrates how user data are transformed into the required features, and Figure 3 provides the overall architecture of the proposed MIND model.

## Methods

### Task Definition

Let a user’s collection of posts on a social media platform be denoted as Pᵢ = {pᵢ₁, pᵢ₂, …, pᵢ_L_}, with each post associated with a time stamp, forming a corresponding set of posting time stamps Tᵢ = {tᵢ₁, tᵢ₂, …, tᵢ_L_}. Each user is labeled according to their psychological state y_i_ (depressed or control).

In TRD tasks, models typically leverage all available user history to predict psychological states. By contrast, the ERD task is defined as a sequential prediction process, where posts are ordered chronologically, and each post is associated with a time step j ( j = 1, 2,…, L). At each time step j (where j < L), the model processes available posts P_i_^j^ = {pᵢ₁, pᵢ₂, …, pᵢⱼ} and time stamps T_i_^j^ = {tᵢ₁, tᵢ₂, …, tᵢⱼ} to generate a prediction ŷ_i_^j^. This task aims to identify the user’s depressive state as early as possible (ie, at the earliest possible time step j ∈(1,L)), with only one final prediction ŷ^j^ allowed. The evaluation metric emphasizes whether the model can make an accurate prediction at an earlier step rather than waiting for the complete history.

### CAD Module

To capture the longitudinal behavioral patterns and temporal regularity of users, we propose a CAD module. This module transforms discrete posting time stamps into a continuous representation of activity density, serving as a robust digital proxy for the user’s temporal activity patterns and circadian stability.

### Activity Rhythm Initialization

We discretize the 24-hour daily cycle into M=768 time slots (same as the Bidirectional Encoder Representation from Transformers (BERT) output dimension), yielding a temporal resolution of approximately 1.875 minutes per slot. This choice of granularity is motivated by two factors: first, it ensures architectural compatibility for efficient feature fusion with BERT-derived embeddings; second, from a clinical perspective, this high-resolution discretization allows the model to capture subtle and transient fluctuations in nighttime activity patterns, which are often critical indicators of depressive behavioral shifts that coarser temporal scales might overlook. To model the natural diffusion of activity around a specific posting event, we use a Gaussian distribution assumption. For computational efficiency, rather than calculating Gaussian values for every time stamp, we implement a cyclic shift mechanism.

We first initialize a fixed base activity vector v_base_∈R^M^ derived from a standard Gaussian distribution (N(μ=0 and σ=1)). σ is set to 1 for ease of computation and regularization, as subsequent influence factors will adjust the distribution dynamics anyway. The distribution covers the interval (–384 to 383), centered at index c_base_=384. Here, σ is a fixed hyperparameter defining the temporal spread of activity associated with a single post. For a post occurring at minute m_n_∈(0,1440), we determine its target slot index as 
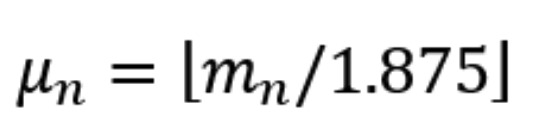
. The individual activity vector is then generated by performing a cyclic left shift on v_base_ by 
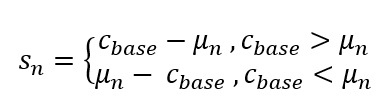
 and 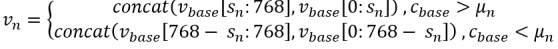. This rotation ensures that the peak of the Gaussian distribution aligns precisely with the user’s posting time on the 768-dimensional grid, regardless of the absolute time zone.

### Adaptive Influence Factor Calculation

We posit that the clinical significance of a post varies depending on when it was posted and what was said. We introduce a composite weighting factor α_n_ to modulate the intensity of each activity vector:

(1) Hourly influence (α_n_^time)^ to capture the inherent importance of specific periods, we use a learnable lookup table. The 24 hours of a day are mapped to a learnable embedding matrix E∈R^24×1^, which is initialized randomly and updated during training. For a post in an hour h_n_, the factor is 
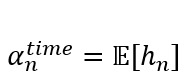
.

(2) Textual influence (α_n_^text^) to incorporate psychological intensity, the post text is encoded into a semantic vector h_text_∈R^384^ via the paraphrase-MiniLM-L6-v2 model. A trainable linear layer then projects this embedding to a scalar influence score α_n_^text^∈R 
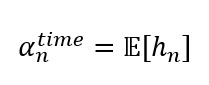
.

### Aggregation

The final representation SC_j_∈R^768^ for a user at time step j is the accumulated weighted activity across all historical posts (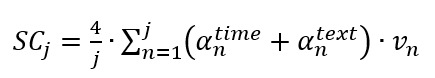). The factor (4/j) is a simple normalization term used to prevent numerical overflow during the accumulation of the SC vector and provides minimal performance gain.

By aggregating these weighted Gaussian kernels,SC forms a dense, continuous waveform. This representation effectively captures the regularity, intensity, and shifts in the user’s daily rhythms. Crucially, by focusing on individual temporal consistency rather than absolute wall-clock time, this formulation remains robust to time zone variations across the global Reddit (Reddit Inc) population.

### LDP Analysis

To overcome rigidity and lack of interpretability in traditional text filtering, we redesign the depression-text selection process by leveraging LLMs with few-shot and zero-shot learning paradigms [26-29]. The emotion categories used in the Rank LLM prompt were designed to conceptually align with symptom dimensions described in established depression screening instruments such as the Patient Health Questionnaire–9 (PHQ-9) and the Beck Depression Inventory. Rather than replicating questionnaire items verbatim, we abstracted common symptom constructs (eg, anhedonia, hopelessness, suicidal ideation, fatigue, appetite disturbance, and guilt) into concise semantic descriptors suitable for LLM-based text scoring. The corresponding prompt is provided in Textbox 1. Importantly, the LLM scoring is not intended as a clinical diagnosis, but as a weakly supervised semantic proxy for depression-related symptom expression in natural language. We further implemented a zero-shot large language model (LLM) prompt for psychological issue detection (Textbox 2).

**Prompt for a large language model (LLM).**

**Prompt for rank large language model (LLM):**

You are a psychologist who needs to assess whether a patient’s text contains depression-related emotions in order to score based on the severity of emotions. Don’t explain why, just output the result.

emotion: {sad, hopeless, failure, suicide, expected to be punished, guilty, cry, lose interest, worthless, low energy, change pattern, irritable, change appetite, tired}

response: 0~5

<example>

**text**: Just it looks so lonely

**score**: 3

**text**: I still feel awful whenever I make a mistake

**score**: 5

</example>

{input text}

**Prompt for analysis LLM:**

You are a clinical psychologist. Read the following text and identify any potential psychological issues.

–Your reply must be very brief (one sentence or less).

–Do not explain your reasoning.

–If no issues are found, reply exactly “None.”

{input text}

**Large language model (LLM) prompt for analysis (zero-shot).**

**System:**

- You are a clinical psychologist.

**User:**

- Read the following text and identify any potential psychological issues:
- Your reply must be very brief (one sentence or less).
- Do not explain your reasoning.
- If no issues are found, reply exactly “None.”

**Text:**

- My job is unstable. It’s fine now, but what about the future?

**Response:**

- Potential issues with stress or anxiety related to work hours.

**Large language model application programming interface** (**API):**

- Due to regional restrictions, the experiments used a third-party API proxy. Based on communication with the provider, the default parameters were fixed at temperature = 1.0 and top_p=1.0 to ensure stability and consistency.

Specifically, the Rank LLM first assigns a depression-related score to each post P_i,j_^topk^=p_top1,_ p_top2_,…, p_topk_. Under the guidance of a small number (2) of expert-labeled examples, each post is scored from 0 to 5, and the top-*k* posts with the highest scores are selected. These selected posts are then analyzed by the Analysis LLM, which generates short free-text descriptions of potential depression-related factors. Each description represents a latent psychological issue inferred from the post (eg, hopelessness, self-harm ideation, or emotional instability). The resulting factor set is denoted as F_i,j_^topk^=f_top1,_ f_top2_,…, f_topk_.

To encode both the selected posts and the generated factors, we use a pretrained text encoder, denoted as encoder. In this work, an encoder is implemented using BERT, but the design allows replacing it with other pretrained language models to enable future improvements without changing the overall framework. While the BERT has a maximum input length of 512 tokens. To ensure stable processing, each post is truncated to the first 512 tokens before being fed into the encoder. Each text sequence is tokenized and fed into the encoder independently, which outputs contextualized token-level representations. For the factor texts, the encoder output is 
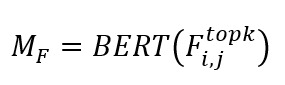
, where M_F_∈R^k×seq×dim^, k is the number of selected posts, L is the sequence length, and dim is the hidden dimension of the encoder. Similarly, the selected posts are encoded as an 
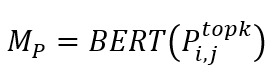
, where M_P_∈R^k×seq×dim^, k is the number of selected posts, L is the sequence length, and dim is the hidden dimension of the encoder.

These matrices are not directly reduced to fixed-length vectors. Instead, we use a cross-attention mechanism to allow the depression factors to guide the representation of the texts. Specifically, MT is used as the query, and MF is used as the key and value (
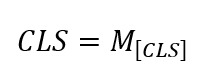
), where M∈R^k×seq×dim^, k is the number of selected posts, L is the sequence length, and dim is the hidden dimension of the encoder. W∈trainable R^dim×dim^.

The cross-attention output is a contextualized representation that integrates both the semantic content of the posts and the depression-related factors inferred by the LLM. After cross-attention, we extract the hidden state corresponding to the CLS token as the final representation of each selected post, as in 
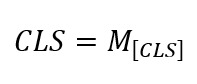
, where CLS∈R^k×dim^, k is the number of selected posts, and dim is the hidden dimension of the encoder.

At this point, we have obtained the user depression-text profile feature CLS enhanced by the LLM. Additionally, to ensure that the historical information posted by the user is not overlooked by the model, we average the embeddings of all the user’s texts and use this as the coarse-grained historical text feature (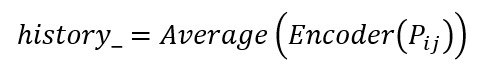 and 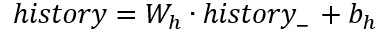). Where history_∈R^384^, history∈R^384^, and W_h_∈trainable R^768×384^ represent the corresponding feature vectors and parameter matrix with specific dimensions.

### Text-Sleep Fusion

First, for sleep-aware text feature enhancement, we leverage the cross-attention mechanism and use temporal activity patterns features as prior knowledge to guide the fusion of depressive text profile features and historical information features, thereby achieving the goal of targeted text feature enhancement as 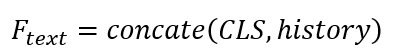, 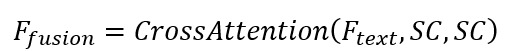, and 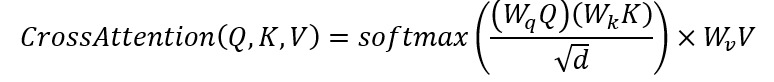), where F_text_, F_fusion_∈R^k+1×dim^, W_q,k,v_∈trainable R^dim×dim^ represent the corresponding feature vectors and parameter matrix with specific dimensions.

Next, to preserve the order of text and distinguish between historical texts and filtered texts, we add trainable positional encoding. The resulting features are then concatenated with temporal activity patterns SC and input into the transformer encoder as 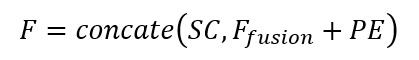 and 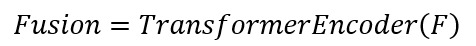, where F∈R^k+2×dim^ and PE∈trainable R^k+2×dim^ represent the corresponding feature vectors and parameter matrix with specific dimensions. As global prior knowledge, temporal activity patterns features SC are inherently different from the sequential attribute of text sequences due to their time-independence; therefore, SC is excluded from positional encoding to ensure the model can correctly distinguish between the two. After in-depth data mining by the transformer encoder, more accurate multimodal features are obtained.

To better address the information compression issue when mapping multimodal features to a single user representation, we adopt the mixture of experts (MoE) approach—a framework originally proposed by Jacobs et al [30] that has recently reemerged in LLM research. By dynamically selecting expert combinations, MoE enables more flexible feature fusion and avoids potential information loss caused by traditional pooling operations, making it well-suited for the demands of contemporary multimodal and large-scale model scenarios. Finally, the final prediction is generated through a linear layer as provided in 
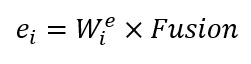
, 
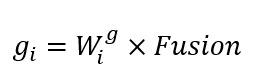
, 
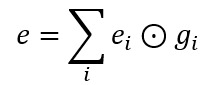
, and 
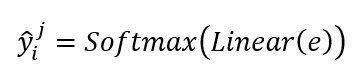
, where e, e_i_ ∈R^dim^, g_i_∈R, W^e^∈trainable R^dim×dim^, and W^g^∈trainable R^dim×1^ represent the corresponding feature vectors and parameter matrix with specific dimensions. Since this is a classification task, we use the traditional cross-entropy loss function, defined as 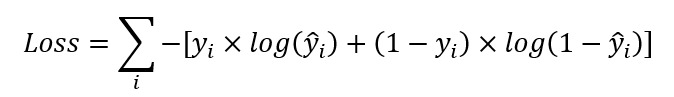. We intentionally did not adjust the class imbalance by reweighting the loss function because the original class distribution reflects the real-world prior probability in ERD tasks. Reweighting the loss may increase false positives and harm early warning precision.

### Sequential Prediction Strategy

The ERD task requires the model to make predictions as user posts arrive over time. Therefore, the model performs inference sequentially over time steps. At each time step j, the model receives all posts up to step j and generates a prediction ŷ∈{0,1}. The model continues predicting at each time step until a positive prediction (ŷ=1) is produced. Once the model outputs 1, the inference process stops, and the user is labeled as depressed. The time step j at which the first positive prediction occurs is recorded as the decision time, which is used to evaluate the earliness metric in ERD.

If the model never outputs 1, the final prediction at the last time step is used, and the user is labeled as control. This decision strategy follows the standard ERD evaluation protocol, where only one final decision is allowed for each user, and earlier correct predictions are rewarded.

### Responsible Deployment Framework

To ensure responsible deployment, several safeguards should be maintained in practice. First, model outputs should serve only as preliminary risk signals. Any high-risk identification must be reviewed by qualified professionals or trained moderators before action is taken. Second, predictions should not trigger account suspension, content removal, or other restrictive actions without human verification. The system is intended to support assistance-oriented interventions rather than enforcement. Third, when feasible, users should be informed that automated risk monitoring systems are in place. The interpretability mechanisms provided by temporal activity patterns and LLM-generated factors may assist human reviewers in understanding the basis of risk signals. Fourth, only publicly available data are processed, and storage duration is limited. Fifth, the model should not replace professional mental health assessment; its role is limited to identifying behavioral patterns potentially associated with depression risk and recommending further evaluation where appropriate.

### Ethical Considerations

The proposed MIND model is designed as a risk estimation framework rather than a diagnostic system. It does not infer clinical diagnoses and should not be used as a stand-alone decision-making tool in medical or administrative contexts. The model should not replace professional mental health assessment; its role is limited to identifying behavioral patterns potentially associated with depression risk and recommending further evaluation where appropriate. Only publicly available data are processed, and storage duration is limited. In real-world deployment, stronger anonymization and encryption mechanisms would be necessary. By explicitly distinguishing risk estimation from clinical diagnosis, the framework aims to balance early intervention benefits with ethical responsibility and user autonomy. In accordance with institutional and relevant ethical guidelines, ethical approval and informed consent were not required for this study, as only publicly available data were used and no human participants were involved. Privacy and confidentiality considerations were not applicable.

## Results

### Dataset

We conducted experiments on the eRisk 2017 dataset, the standard benchmark for ERD tasks [20]. The dataset contains 137 depressed users and 755 control users, divided into training (486) and testing (406) sets, collected from the Reddit platform. Depressed users were identified through self-reports in depression-related forums, with explicit confirmation of clinical diagnosis. Control users were sampled from nondepression subforums and depression subforums where users exhibited no depressive symptoms.

To ensure privacy, all user IDs were anonymized, and only text content and posting times were preserved. Table 1 summarizes the dataset statistics.

**Table 1:** Dataset statistics.

Characteristic	Train (depression)	Train (control)	Test (depression)	Test (control)
Users	83	403	52	349
Posts	30,851	172,834	18,706	217,665
Average posts per user	371.7	655.5	359.7	623.7

The dataset does not provide user time zone information, and all time stamps are recorded in platform time. Therefore, the proposed method does not rely on absolute clock time, but instead models the relative distribution of posting activity within each user’s history. Because the representation is learned from long-term aggregated activity, it reflects stable temporal patterns rather than specific clock-based sleep schedules.

### Implementation Details

The experiments were conducted via a third-party application programming interface (API) proxy service (due to regional access restrictions), which implemented a preconfigured environment for model deployment. To ensure system stability and experimental consistency within this framework, we used the default decoding parameters as confirmed by the service provider: temperature = 1.0 and top_p = 1.0. Other auxiliary parameters were maintained at their default settings to ensure the reproducibility of the scoring results under the same API environment. The encoder was implemented with BERT [31], and embeddings were generated using the frozen paraphrase-MiniLM-L6-v2 model. In the CAD module, the Gaussian parameter σ was set to 1. All attention layers adopted multihead attention with 8 heads.

In the text-sleep fusion module, the transformer encoder was configured with 4 layers. Training was performed using the Adam optimizer with a learning rate of 2e-5, a batch size of 4, and 7-14 epochs. Early stopping was applied based on validation performance, where training was terminated if the validation metric did not improve for 3 consecutive epochs. Training was conducted on NVIDIA A30 graphics processing units (GPUs) with PyTorch (version 2.3.0; Meta AI), supported by an AMD EPYC 7543 CPU and 64 GB memory. To mitigate randomness, 5 random seeds were used.

### Comparison Methods

We compared MIND with multiple baselines, including official dataset baselines and subsequent studies. “Logistic regression” uses term frequency–inverse document frequency features and logistic regression, reframing ERD as multiclass classification [32]. “Feature-Rich” methods rely on feature engineering, including topic extraction using latent Dirichlet allocation and user-level statistical features [33]. “BiLSTM+Attention” models sequence information from the full user history using LSTM networks with attention mechanisms [16]. The “risk window” approach applies a sliding-window strategy to trigger warnings when depressive posts appear consecutively [16]. The “τ-SS3” model uses an enhanced n-gram-based approach for dynamic phrase sequence detection [34]. The “early list” introduces reinforcement learning-based gating to encourage early predictions [12]. “HAN-Psych” uses hierarchical attention networks inspired by clinical questionnaire structure [15]. Finally, “ESDM” balances earliness and accuracy using a loss function combining LSTM and linear attention mechanisms [13].

For fairness, all models followed their original decision-making rules. For methods without explicit rules, we adopted the default “diagnose-as-soon-as-identified” strategy.

Due to GPU memory constraints, the original implementations of ESDM and EarlyList require substantially higher computational resources. Therefore, in our experiments, these 2 methods were limited to processing the most recent 64 posts per user. In the ERD setting, later posts usually contain stronger depressive signals, so this restriction does not disadvantage the baselines and may even simplify the prediction task. We also verified that the performance obtained under this setting is consistent with the results reported in the original papers. This constraint mainly affects runtime measurement; therefore, the inference time of these methods is not reported for fair comparison. The inference time of τ-SS3 is difficult to compute and is not discussed in this paper.

### Metrics

ERD introduces a specialized evaluation metric balancing “earliness” and “accuracy.” This metric penalizes late predictions exponentially beyond a certain time threshold, ensuring models are rewarded for early and correct predictions, as provided in 
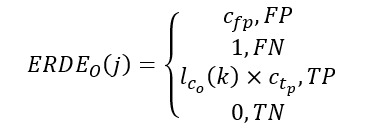
 and 
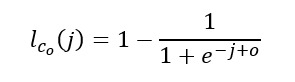
, where l_co_(j)∈[0,1], c_fp_=positive/total.

In addition, we also introduced the *F*_1_-score of the final early warning results for the ERD task (*F*_1ERD_) and the *F*_1_-score for the TRD task (*F*_1TRD_) respectively, to more accurately evaluate the model’s accuracy and its generalization ability on the TRD task. Overall, a superior model is characterized by smaller values in metrics ERDE_5_ and ERDE_50_, and larger values in metrics *F*_1ERD_ and *F*_1TRD_.

### Evaluation Outcomes

Tables 2 and 3 show that our proposed MIND model consistently outperforms all baseline models across all key metrics. Specifically, based on the best-performing runs reported in Table 3, MIND achieves a relative improvement of 25.4% in ERDE_5_ and 10.7% in ERDE_50_ compared to the strongest baseline. Furthermore, to address the potential bias of single-run results, we also evaluated the average performance across 5 random seeds. As shown in Table 3, the model maintains a robust advantage with mean improvements of 22% (ERDE_5_) and 13.1% (ERDE_50_), respectively. This demonstrates that the performance gain of MIND is not a result of stochastic variance but represents a systemic enhancement in early depression detection. It can be observed that two of the *F*_1_-scores achieve only slight improvements, while the increased inference time is mainly attributed to the introduction of LLMs. We also present the Cohen *d* comparison results between our model and the 2 current state-of-the-art models in Table 4.

**Table 2:** Best-performing metrics across 5 random seeds.

Model	↓ERDE_5_^a^	↓ERDE_50_	↑*F*_1ERD_^b^	↑*F*_1TRD_^c^	↓Time cost (s)
LR^d^	13.70	8.49	40.50	60.20	4710
Feature-Rich	13.10	8.45	35.85	63.08	7558
BiLSTM+Attention	12.49	9.63	56.22	62.93	1101
Riskwindow	13.01	9.79	60.61	62.90	920
τ-SS3	13.36	8.60	49.76	54.69	—^e^
EarlyList	16.45	14.87	27.34	17.54	—
HAN-Psych	10.77	8.11	63.19	70.31	1330
ESDM	10.98	7.78	66.23	71.26	—
MIND^f^	8.03	6.94	67.17	72.72	2208

^a^ERDE: early risk detection error.

^b^ERD: early risk detection.

^c^TRD: traditional risk detection.

^d^LR: logistic regression.

^e^Not available.

^f^MIND: monitoring of individual nighttime dynamics.

**Table 3:** Mean (SD) of metrics across 5 random seeds.

Model	↓ERDE_5_^a^	↓ERDE_50_	↑*F*_1ERD_^b^	↑*F*_1TRD_^c^
LR^d^	13.70 (0.00)	8.49 (0.00)	40.50 (0.00)	60.20 (0.00)
Feature-Rich	13.35 (0.23)	8.66 (0.22)	34.68 (1.23)	62.10 (0.68)
BiLSTM+Attention	12.71 (0.40)	10.37 (0.49)	54.87 (1.30)	62.17 (0.80)
Riskwindow	13.57 (0.55)	9.89 (0.21)	58.79 (2.29)	62.36 (0.33)
τ-SS3	13.66 (0.72)	9.25 (0.53)	47.72 (1.50)	53.20 (1.18)
EarlyList	16.97 (0.86)	15.73 (0.63)	26.50 (0.91)	17.19 (0.27)
HAN-Psych	11.27 (0.56)	8.36 (0.43)	60.27 (1.80)	66.59 (2.14)
ESDM	12.37 (0.99)	8.81 (0.95)	58.81 (4.50)	66.03 (3.05)
MIND^e^	8.79 (0.67)	7.26 (0.44)	64.54 (2.80)	69.57 (3.44)

^a^ERDE: early risk detection error.

^b^ERD: early risk detection.

^c^TRD: traditional risk detection.

^d^LR: logistic regression.

^e^MIND: Monitoring of Individual Nighttime Dynamics.

**Table 4:** Comparison of Cohen d metrics between Monitoring of Individual Nighttime Dynamics (MIND) and SOTA baselines.

Model	ERDE_5_^a^	ERDE_50_	*F*_1ERD_^b^
HAN-Psych	3.998	2.719	1.800
ESDM	4.217	2.198	1.527

^a^ERDE: early risk detection error.

^b^ERD: early risk detection.

We computed the Cohen *d* values between the state-of-the-art models and our MIND model on the ERD task based on experimental results from 5 random seed runs, so as to quantify the statistical significance of performance improvements achieved by our model.

### Ablation Study

To evaluate each component’s contribution, we performed ablation experiments. The “Sleep” variant removed the CAD module, relying solely on text. The “Text” removed the LLM depression profiler, using only temporal activity patterns features. The “LLM” variant replaced LLM-based filtering with the previous method, the template matching-based approach. The “History” variant removed historical text features. The “Fusion” variant removed cross-attention and MoE, using only a transformer encoder.

Table 5 presents the results of our ablation experiments. It can be observed that removing the CAD has a significant impact on the model; furthermore, the CAD alone struggles to achieve favorable performance. Replacing the LLM component in the model with existing methods exerts a substantial influence on the experimental results. Removing user historical features has a more pronounced effect on ERDE_5_, which is more sensitive to earliness, but a relatively minor impact on ERDE_50_, which has a higher tolerance for earliness. Removing the cross-attention and expert gating mechanisms only has a slight impact on the overall performance of the model.

**Table 5:** Results of the ablation study.

Model	↓ERDE_5_^a^	↓ERDE_50_	↑*F*_1ERD_^b^
MIND^c^	0.080	0.069	0.671
Sleep	0.094	0.078	0.633
Text	0.153	0.122	0.430
LLM^d^	0.103	0.093	0.655
History	0.106	0.078	0.668
Fusion	0.084	0.071	0.669

^a^ERDE: early risk detection error.

^b^ERD: early risk detection.

^c^MIND: Monitoring of Individual Nighttime Dynamics.

^d^LLM: large language model.

### Case Study

We visualize temporal activity patterns in heatmaps for depressed and control users. Control users (A) typically show near-zero activity between 6 AM and 11 AM, indicating sufficient and regular sleep. In contrast, depressed users (B) display irregular activity across all hours, often engaging in late-night posting, with loosely defined sleep windows (eg, 5 AM to noon).

Table 6 shows examples of LLM scoring and analysis. These examples highlight MIND’s interpretability: high-risk posts (eg, self-harm) are prioritized, while latent depressive tendencies are also captured, providing actionable insights for clinical support.

**Figure 4:**
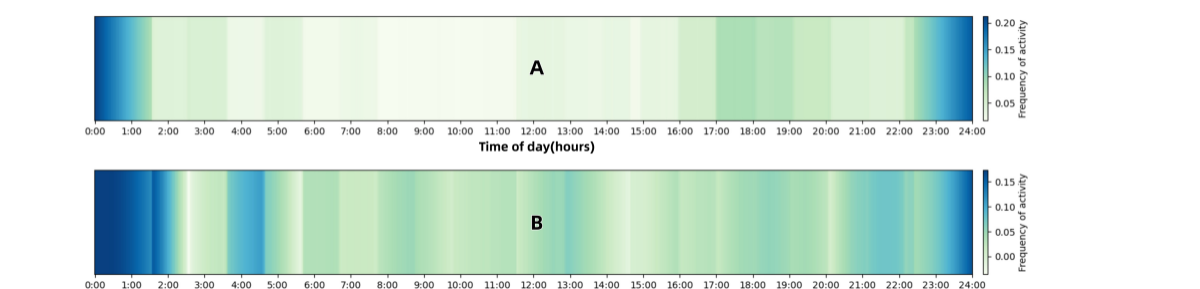
Visualization of temporal activity patterns in depressed vs control users.

It should be noted that due to the lack of time zone information in the dataset, direct quantitative comparisons of absolute posting times (eg, mean active hour) are not statistically robust. Instead, our analysis of the heatmaps in Figures 4A and B focuses on relative activity rhythms. We specifically look for indicators of circadian rhythm disruption, such as fragmented activity during typical rest periods and the lack of a stable, continuous inactivity window. These patterns provide more reliable clinical signals for depression than absolute temporal metrics.

**Table 6:** Examples of large language model ranking scores and corresponding depression factor analysis.

Score	User post	Depression factor
5	Today is a bad day, maybe I should cut my wrist.	Potential self-harm ideation
2	My job is unstable. It’s fine now, but what about the future.	Potential stress or anxiety related to work hours.
1	I tried to express this meaning without directly accusing OP of abusive behavior.	Potential fear of confrontation or difficulty addressing conflict directly.
0	I always thought this was why boys urinate standing up.	None

### Contribution Analysis

**Figure 5:**
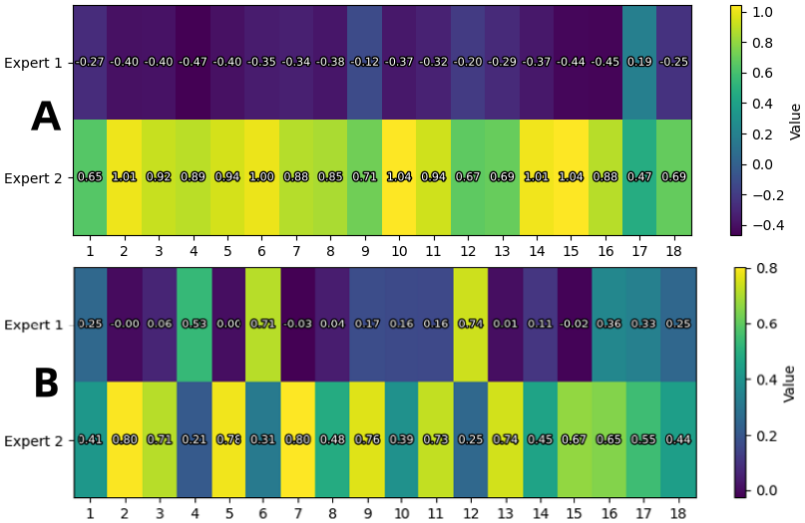
Heatmaps of feature contribution analysis.

We visualized the contribution degree of different features to the final classification. Column 1 corresponds to the user’s temporal activity patterns, Column 18 corresponds to the historical features, and Columns 2-17 correspond to the texts with the strongest depressive state among the texts posted by users. “Expert” represents the contribution degree of the expert model in Moe to the result. A higher contribution to the final discrimination result is indicated by a larger number at the corresponding position and a color that leans more toward yellow, while a lower contribution is shown by a smaller number and a color that leans more toward dark blue. As shown in Figure 5, Expert 1 is sensitive to the features of depressed users, whereas Expert 2 is more sensitive to those of control users (Figure 5A and 5B). Although temporal activity patterns and historical features have a relatively stable contribution ratio to the discrimination, the text features of users contribute the most to the final result.

### Reduction-Based Validation

**Figure 6:**
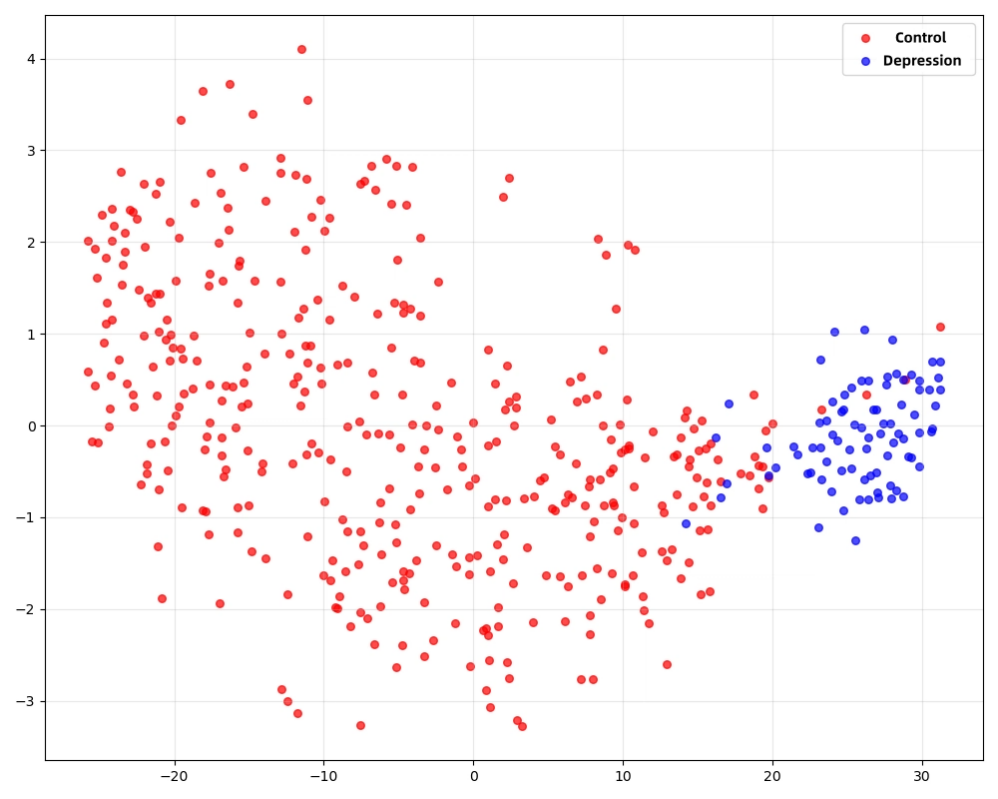
Dimensionality reduction visualization of the distinction between depressed and control users.

This subsection presents the visualization of user features mapped in a 2D space, where the features have been processed using our proposed method. For dimensionality reduction, we adopted t-distributed stochastic neighbor embedding [35] and principal component analysis [36]. Specifically, features of depressed users are displayed in blue, while those of nondepressed users are shown in red. As illustrated in Figure 6, the features of depressed users are clustered in a distinct region. Meanwhile, the features of most nondepressed users are far apart from those of depressed users; in contrast, the difference between the features of a small number of nondepressed users and those of depressed users is not significant.

### Hyperparameter Analysis

**Figure 7:**
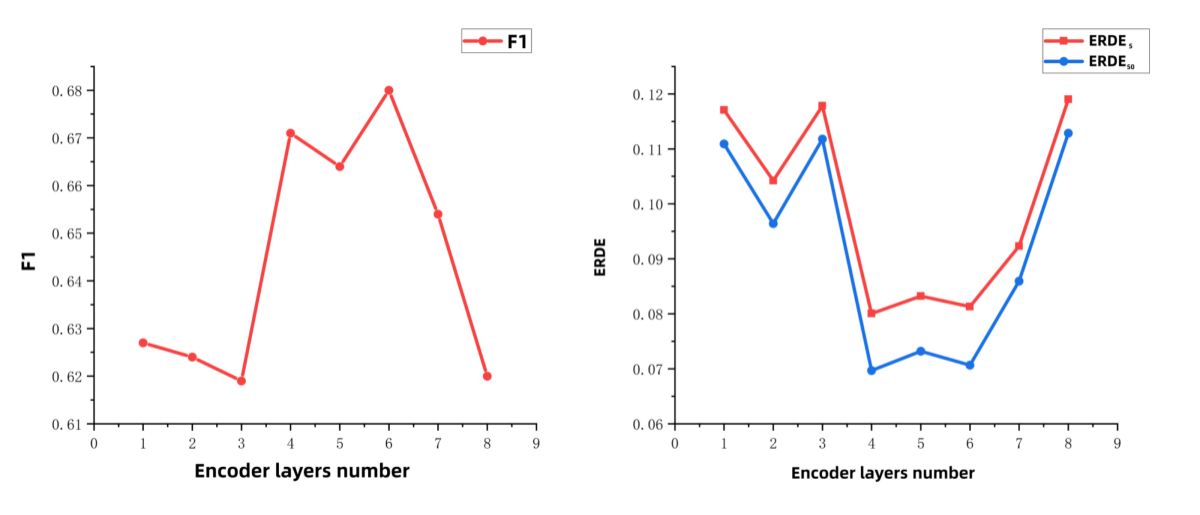
Performance changes of the model in terms of F1-score and early risk detection error (ERDE) as model parameters vary.

The most computationally intensive component of the entire model is the encoder layer. To strike a balance between computational efficiency and accuracy, we conducted experiments on the selection of the number of encoder layers. As shown in Figure 7, performance changes of the model in terms of *F*_1_-score and early risk detection error as parameters vary. When the number of encoder layers is between 3 and 5, the early risk detection error values reach their minimum, while the *F*_1_-score also peaks in a similar range. Within this range, increasing the number of layers yields limited improvement in model performance; thus, we selected 4 layers, which offer the most efficient computation speed.

## Discussion

### Overall Analysis

The previous section presented the performance of MIND on the eRisk 2017 dataset, along with results from ablation studies, case analyses, contribution visualization, and reduction-based validation. Overall, our findings demonstrate that the proposed approach consistently outperforms existing methods across evaluation metrics, except for higher inference-time costs. In this section, we provide a detailed analysis of each module’s strengths and limitations.

### CAD Analysis

In the ablation experiments, removing the CAD module resulted in a 17.5% increase in ERDE_5_ and a 13% increase in ERDE_50_, while *F*_1ERD_ decreased by 5.6%. These results indicate that CAD’s primary contribution lies in enhancing earliness, with a relatively smaller impact on accuracy.

Although the introduction of temporal activity patterns modeling provides a novel modality for ERD, the CAD module alone yields limited performance, confirming that text features remain the dominant modality, while CAD serves as an auxiliary enhancement. This observation aligns with the contribution analysis, where temporal activity patterns provided stable contributions but were not the most decisive factor compared to the text feature.

Case studies showed that temporal activity patterns clearly distinguished depressed from nondepressed users. Depressed users exhibited irregular, insufficient temporal activity patterns, while nondepressed users displayed consistent sleep cycles. Such visualization not only aids classification but also offers clinicians interpretable evidence for interventions. However, the approach has limitations: since temporal activity patterns are inferred from aggregated posting time stamps, the model only captures coarse-grained, long-term sleep habits. While effective for users with persistent depressive tendencies, it is less sensitive to short-term depressive episodes, underscoring the need for finer-grained integration with contextual textual analysis.

### Clinical Alignment With Depression-Related Sleep Disturbances

Extensive clinical research has established sleep disturbance as a core feature of major depressive disorder. Patients with depression frequently exhibit insomnia, early morning awakening, sleep fragmentation, circadian rhythm instability, shortened rapid eye movement latency, and altered sleep architecture [21-24].

Although the proposed CAD module does not measure physiological sleep architecture, the extracted temporal activity patterns may reflect behavioral correlates of these clinically observed abnormalities. For example, early morning awakening may correspond to premature termination of low-activity intervals in posting behavior; sleep fragmentation may manifest as increased nighttime posting density; and circadian rhythm instability may be reflected by higher variance in inferred rest-activity windows across days.

Therefore, the CAD representation should be interpreted as a digital behavioral proxy of circadian irregularity rather than a direct physiological sleep measurement. This alignment strengthens the clinical interpretability of the model while remaining within the scope of computational social media analysis.

### LDP Analysis

Replacing the LDP module with template matching in ablation studies caused the ERDE_5_ to increase by 28.7% and ERDE_50_ by 34.7%, with a 2.3% decrease in *F*_1ERD_. This demonstrates that the LLM component substantially improves early detection capability, although gains in accuracy are more modest.

One of the most significant benefits of LDP is its improvement in interpretability. Unlike static filtering, the LLM not only identifies depressive posts but also traces underlying risk factors (eg, hopelessness and suicidal ideation). This provides actionable insights into users’ depressive triggers and key posts.

However, performance was found to be sensitive to the choice of base model. During experiments, different LLMs yielded varying results, with GPT-4o (OpenAI) showing the most balanced performance. This suggests that model selection and optimization remain critical directions for future research.

Moreover, reliance on external APIs and longer inference time limits scalability. Deploying locally optimized LLM could address both efficiency and privacy concerns. Historical features, while less impactful overall, contributed significantly at earlier time steps, indicating their importance for very early detection.

### TSF Analysis

The TSF module was designed to align and integrate multimodal features. Ablation experiments confirmed its role: removing TSF increased the ERD metric by 9.16% and earliness by 7.2%, although *F*_1_ remained stable (fluctuations <1%).

This demonstrates that TSF consistently enhances early warning performance while maintaining accuracy. Its lightweight fusion process effectively balances multimodal collaboration without incurring significant overhead.

Further analysis revealed that when only text features were available, TSF offered no clear advantage over a simple transformer encoder. This suggests that its benefit primarily arises from the cross-modal alignment between text and temporal activity patterns. Improving TSF’s generalization ability to text-only scenarios is a potential avenue for future improvement.

### Computational Cost of the LDP

As provided in Table 3, our model exhibits a relatively noticeable increase in time consumption, with an additional 878 seconds compared to the existing state-of-the-art models. The LDP introduces additional computational overhead due to repeated API calls. For each user, the Rank LLM evaluates all posts, and the Analysis LLM processes the top-*k* posts (k=16). In the eRisk2017 dataset, the average number of posts per user is approximately 419. Thus, each user requires about 419 Rank LLM calls and 16 Analysis LLM calls. The experiments were conducted using an online LLM API service. Because the API server is located overseas, all requests must be sent through a virtual private network proxy, which introduces significant network latency. In addition, the experimental environment processes requests sequentially, without multithreading or batching. Under this setting, the average time cost for processing a single post (including network communication and API response) is approximately 1.7-2.5 seconds. It should be noted that most of the time, overhead comes from network communication rather than model inference itself. In practical deployment, API calls can be parallelized and executed without virtual private network routing, so the actual runtime can be significantly lower than in the experimental environment.

### Deployment and Generalization

The core method of this paper, CAD, aims to analyze users’ sleep patterns through the timing of their posts, enabling earlier warning of users’ depressive states. In practical deployment, platforms can obtain more detailed user information—not only time stamps of users’ posting behavior but also those of their reading behavior. This allows the model to generate more accurate temporal activity patterns, thereby improving performance.

The proposed method should achieve favorable results on most social media platforms. However, it is difficult to generalize to platforms focusing primarily on visual content, such as Instagram (Meta Platforms). This is because the method takes textual information as the primary input and temporal activity patterns as an auxiliary feature to predict user depression.

Regarding generalization to other mental disorders, the method proposed in this paper cannot be directly applied. This is due to the lack of authoritative and widely accepted evidence linking other mental illnesses to sleep patterns, which requires further research.

Because the LLM component operates via explicit prompt instructions rather than model-specific fine-tuning, the framework is model-agnostic. Future researchers can substitute GPT-4o with other LLMs by preserving the prompt structure and scoring scale, facilitating adaptation as LLM technologies evolve.

### Limitations

Due to GPU memory limitations, 2 baselines (ESDM and EarlyList) were evaluated using only the most recent 64 posts per user. Although this setting follows the practical constraints of the original implementations and does not negatively affect performance in the ERD scenario (where later posts usually contain stronger signals), it may still introduce minor differences compared with fully unrestricted evaluation. Future work will include experiments under larger computational resources to further verify fairness across all baselines.

The proposed CAD module models long-term temporal activity patterns derived from posting time stamps rather than actual sleep behavior. Reduced posting activity does not necessarily indicate sleep, as it may also result from work schedules, social engagement, travel, or decreased platform usage. Therefore, the extracted features should be interpreted as proxies of rest–activity regularity rather than direct measurements of sleep-wake rhythms.

### Conclusions

In this study, we proposed a novel method for early risk detection of depression on social media, integrating temporal activity patterns modeling and LLM-based text filtering and analysis. Unlike prior work, which exclusively relies on text features for depressive state analysis, our approach incorporates posting time stamps to infer users’ temporal activity patterns and leverages LLM to improve upon traditional template-matching methods, thereby constructing a more accurate profile of depressive texts.

Experiments on the eRisk 2017 benchmark demonstrate that MIND not only achieves superior performance across multiple evaluation metrics, despite higher inference costs, but also provides enhanced interpretability (via temporal activity patterns visualization and LLM-based tracing of latent depressive factors). The temporal activity patterns and the LLM’s identification of latent depressive factors allow the model to trace back depressive risk signals, offering strong support for clinical intervention and treatment.

Overall, MIND establishes a new paradigm for multimodal ERD by combining temporal activity patterns and advanced language modeling, breaking the limitation of prior text-only methods—with high practical value for real-world applications in mental health monitoring.

## Data Availability

Our research on depression risk may raise ethical concerns. The data used in this study come from a publicly available dataset shared by other researchers. To protect user privacy, all social media data underwent a strict anonymization process conducted by the dataset providers before usage. We followed all relevant ethical guidelines and legal requirements to ensure that no privacy violations occurred during the research process. The proposed model is not intended as a diagnostic tool but rather as a risk estimation framework to support monitoring and early intervention. All textual examples shown in this paper were paraphrased by the authors to remove sensitive information and do not represent original user posts.

## Abbreviations

BDI: Beck Depression Inventory
BERT: Bidirectional Encoder Representation from Transformers
CAD: circadian activity dynamics
ERD: early risk detection
GPU: graphics processing unit
LDP: LLM depression profiler
LLM: large language model
MIND: Monitoring of Individual Nighttime Dynamics
MoE: mixture of experts
PHQ-9: Patient Health Questionnaire–9
TRD: traditional risk detection,
TSF: text-sleep fusion
